# Prevalence of Pulmonary Tuberculosis among Adults in a Rural Sub-District of South India

**DOI:** 10.1371/journal.pone.0042625

**Published:** 2012-08-15

**Authors:** Vineet K. Chadha, Prahlad Kumar, Sharada M. Anjinappa, Sanjay Singh, Somashekar Narasimhaiah, Malathi V. Joshi, Joydev Gupta, Lakshminarayana, Jitendra Ramchandra, Magesh Velu, Suganthi Papkianathan, Suseendra Babu, Hemalatha Krishna

**Affiliations:** 1 Epidemiology and Research Division, National Tuberculosis Institute, Bangalore, Karnataka, India; 2 Office of the Director, National Tuberculosis Institute, Bangalore, Karnataka, India; 3 Monitoring and Evaluation, National Tuberculosis Institute, Bangalore, Karnataka, India; 4 Human Resource Development, National Tuberculosis Institute, Bangalore, Karnataka, India; 5 Laboratory, National Tuberculosis Institute, Bangalore, Karnataka, India; McGill University, Canada

## Abstract

**Background:**

We conducted a survey to estimate point prevalence of bacteriologically positive pulmonary TB (PTB) in a rural area in South India, implementing TB program DOTS strategy since 2002.

**Methods:**

Survey was conducted among persons ≥15 years of age in fifteen clusters selected by simple random sampling; each consisting of 5–12 villages. Persons having symptoms suggestive of PTB or history of anti-TB treatment (ATT) were eligible for sputum examination by smear microscopy for Acid Fast Bacilli and culture for *Mycobacterium tuberculosis*; *t*wo sputum samples were collected from each eligible person.

Persons with one or both sputum specimen positive on microscopy and/or culture were labeled suffering from PTB. Prevalence was estimated after imputing missing values to correct for bias introduced by incompleteness of data.

In six clusters, registered persons were also screened by X-ray chest. Persons with any abnormal shadow on X-ray were eligible for sputum examination in addition to those with symptoms and ATT. Multiplication factor calculated as ratio of prevalence while using both screening tools to prevalence using symptoms screening alone was applied to entire study population to estimate prevalence corrected for non-screening by X-ray.

**Results:**

Of 71,874 residents ≥15 years of age, 63,362 (88.2%) were screened for symptoms and ATT. Of them, 5120 (8.1%) - 4681 (7.4%) with symptoms and an additional 439 (0.7%) with ATT were eligible for sputum examination. Spot specimen were collected from 4850 (94.7%) and early morning sputum specimens from 4719 (92.2%). Using symptom screening alone, prevalence of smear, culture and bacteriologically positive PTB in persons ≥15 years of age was 83 (CI: 57–109), 152 (CI: 108–197) and 196 (CI :145–246) per 100,000 population respectively. Prevalence corrected for non-screening by X-ray was 108 (CI: 82–134), 198 (CI: 153–243) and 254 (CI: 204–301) respectively.

**Conclusion:**

Observed prevalence suggests further strengthening of TB control program.

## Introduction

National Tuberculosis Programme (NTP) based on a cost effective operational strategy was implemented all over India from 1962; after a nation-wide survey during 1955–1958 revealed that tuberculosis (TB) was highly prevalent throughout the country [1]. Surveys carried out thereafter in geographically defined areas revealed that the prevalence of TB continued to be high, though varied, in different parts of the country [2]. Taking cues from a review of NTP, Revised National tuberculosis Control Programme (RNTCP) adopting DOTS (an internationally recommended strategy for TB control) was launched in 1997 and expanded in phases to cover the entire population by 2006 [3]. Implementation of RNTCP lead to improvements in case detection and high treatment success rates in most parts of the country [3], [4]. Indeed, a decline of about 50% in prevalence of pulmonary tuberculosis (PTB) was observed from 1999 to 2006 in a sub-division of Thiruvallur district, Tamil Nadu state [5]. Since, this observation pertained to a single geographical site, Government of India identified six institutions to carry out independent surveys in seven other sites (districts/sub-division of a district) located in different geographical regions using a generic protocol. The objective of the survey at each site was to find out the point prevalence of bacteriologically positive PTB among adults, which would provide a baseline data to measure the future trends in different parts of the country by repeat surveys. The personnel to be involved at these sites were trained in actual field conditions at a common site by a team of vastly experienced trainers. One of these surveyed sites was the Nelamangala sub-division in Bangalore rural district, the results of which are reported hereunder.

## Materials and Methods

### Study site and setting

Nelamangala is one of the four sub-divisions in Bangalore rural district. This district is located adjacent to Bangalore city which is the capital of Karnataka state in southern India. DOTS strategy is being implemented in the entire district since 2002. Nelamangala was chosen for this survey so that the trends from the past could also be elicited since a similar survey had been carried out there during the year 1975 [6].

Majority of adults in Nelamangala are engaged in agricultural occupations and a proportion work in factories located in a bordering industrial area in Bangalore city.

### Study population

Persons ≥15 years of age residing in Nelamangala sub-division for ≥6 months comprised the study population.

### Sampling

Simple random sampling (SRS) was adopted for selection of clusters. Each cluster corresponded to a group of villages in the jurisdiction of a ‘Gram Panchayat’ – a local self government consisting of elected members of the community and having responsibility to implement rural development programs. Office bearers of the Panchayats visited during planning phase of the study insisted for political reasons to cover all the villages in their jurisdiction. There were altogether 25 panchyats in Nelamangala sub-division.

Since the exact count of adult population in individual clusters was not known, fifteen (arbitrarily decided number) clusters were selected and the survey was planned to start in the first selected cluster covering all eligible persons and proceed to subsequent clusters in order of their selection till the required sample size was achieved.

### Sample size

Sample size was originally calculated at 47,828 to estimate the prevalence within 20% of the true value at 5% level of significance considering a design effect of 2 to account for cluster sampling. Expected prevalence of bacteriologically positive PTB (positive for AFB on microscopy and/or culture) while using both screening tools – symptom screening and chest X-ray, was arbitrarily considered at 400 per 100,000 population. During the earlier survey in the area in 1975, prevalence of culture positive PTB in the age group of ≥15 years while using both screening tools was 480 per 100,000 population [6]; prevalence of bacteriologically positive PTB was not reported.

Since the mobile X-ray equipment broke down during the course of the survey, sample size was revised during the course of the survey to 68,400 for an expected prevalence of 280 per 100,000 population assuming that 30% of PTB cases would be missed due to non-screening by X-ray. This assumption was based from the data of the previous survey in the same area [6].

### Key variables

Key study variables were: age, sex, presence of pulmonary symptoms, history of anti-TB treatment (ATT), presence of abnormal shadow on X-ray, result of sputum smear microscopy – spot & early morning specimen and result of culture - spot & early morning specimen.

### Data collection instruments

The following schedules were useful for data collection:-

- Household form: to enlist all residents by age and sex
- Individual card, for each eligible person: to record study identification number (ID), age, sex, symptom status, X-ray film number, date of sputum collection, results of sputum smear and culture examination
- Laboratory register to record sputum specimen numbers and results of smear and culture examination
- X-ray result form

### Field Procedures

Field work was carried out during October 2008–June 2010.

A planning visit was made to each Panchayat office and each village to familiarize the officials, village leaders and the community with the purpose and procedures of the survey and to seek their cooperation. In each village, a rough sketch of lanes and hamlets showing approximate number of houses in each was drawn, after going around and in discussions with village leaders. Survey in each village began on a mutually agreed date. Enumerators went to each household and recorded the age, sex and resident status of each individual. Each eligible person (15 years or more in age and residing for ≥6 months in the household or in any other village in Nelamangala sub- division) was registered into an individual card. Subsequently, a symptom elicitor queried each eligible individual for presence of symptoms suggestive of PTB (persistent cough for ≥2 weeks, fever or chest pain for ≥1 month, presence of blood in sputum any time during last 6 months) and history of ATT. Field supervisors re-interviewed 10% of eligible individuals, as a quality control mechanism.

Individuals having pulmonary symptoms suggestive of PTB or a positive history of ATT were eligible for sputum examination. A spot sputum specimen was collected at a temporarily setup sputum collection centre within the village, after briefing by the laboratory technician (LT) on how to bring out good sputum sample and spit into a pre-numbered sterilized screw capped sputum cup. After a satisfactory extraction of spot specimen, a pre-numbered empty bottle was given for collecting another sample next morning. Sputum containing bottles marked with ID of each patient were transported in a sputum box on day of collection to the accredited laboratory of the institute.

In six of the clusters, each registered person was also screened by X-ray. A 70 mm photofluorography film of the chest was taken at the temporarily setup centre using a mobile mass miniature radiography (MMR unit); pregnant women and bed ridden individuals were excluded (screening by X-ray could not be undertaken in other clusters due to breakdown of equipment during the middle period of the survey). After processing of exposed MMR rolls in X-ray laboratory of the Institute, each film was read and classified by two trained independent X-ray readers into one of the categories - N (normal)/TI (technically inadequate)/A (lung pathology other than tuberculosis)/B (tuberculosis inactive)/C (tuberculosis active). Persons with their films labeled as TI/A/B/C by any of the two X-ray readers were eligible for sputum examination, in addition to those having symptoms suggestive of PTB or history of ATT.

### Laboratory procedures

Sputum specimens were subjected to smear microscopy for acid fast bacilli (AFB) and culture for *M. tuberculosis* following standard laboratory procedures, at a national reference laboratory located in National Tuberculosis Institute, Bangalore (NTI) [7], [8].

Two direct smears were made from each specimen on new labeled slides under aseptic conditions in a bio–safety cabinet. Each smear was stained using 0.1% auramine-O and 0.5% potassium permanganate and examined under a fluorescence microscope at a magnification of 200×.

After taking out the amount required for making smears, each specimen was homogenized and transferred to a McCartney bottle. For decontamination, 4% sodium hydroxide was added in a volume twice that of sputum specimen (Modified Petroff's Method) and incubated in a shaker for 20 minutes. Sterile distilled water was then added up to the neck of the bottle and centrifuged at 3000 rpm (revolutions per minute) for 15 minutes. The supernatant was decanted and the deposit was inoculated onto 2 slopes of Lowenstein-Jensen (LJ) medium. Cultures were incubated at 37°C and examined for the presence of mycobacterial colonies every week for 8 weeks. Culture was discarded in case of contamination or no growth at 8 weeks. The growth if observed was subjected to Niacin test and incubation on LJ medium containing p-nitro benzoic acid (PNB) in a concentration of 500 µg/ml. It was labeled as *M. tuberculosis*, if Niacin test was positive and no growth was observed on PNB containing medium.

Quality assurance of sputum microscopy and culture was undertaken using the existing mechanism of RNTCP [8].

### Study investigators

Survey was carried out under the overall supervision of the Senior Epidemiologist in the institute. Enumeration of residents in the households, screening by interview, sputum collection and data entry was undertaken by trained contractual field staff and monitored by experienced institutional field staff. Subjecting the study subjects to MMR, reading of X-ray films, examination of sputum by smear and culture and analysis was undertaken by experienced staff of the institute.

### Ethical considerations

Survey was approved by the Institutional Ethics Committee.

Written consent for participation was sought from each individual, after explaining procedures of the survey and its benefits to the individuals and community, through material printed in local language and also personally by field staff. No one was compelled to participate.

TB cases detected during the survey were counseled and facilitated to initiate ATT at the nearest RNTCP facility. For the patients who failed to reach the RNTCP facility, repeated visits (upto a maximum of nine) were made to their households by the field staff to persuade them and also some times to accompany them to RNTCP facility in order to initiate ATT. Follow up during treatment was the responsibility of routine staff of RNTCP.

Individuals with symptoms but not having TB were advised to seek treatment at the local health center; however no active intervention was undertaken by survey team for referral or otherwise.

Village leaders were informed of the findings of the study emphasizing their role in raising awareness regarding symptoms of TB and availability of quality services under RNTCP.

### Definitions

Smear Positive case: An individual with at least one sputum specimen found to be positive for AFB on smear microscopy, irrespective of culture result.

Culture Positive case: An individual with at least one sputum specimen found to be positive for *M. tuberculosis* on culture, irrespective of smear result.

Bacteriologically positive case: An individual with at least one sputum specimen found to be positive for AFB on microscopy and/or *M. tuberculosis* on culture.

### Statistical methods

Digitized data was verified and analyzed using SPSS version 17.0 and STATA version 12.0.

Point prevalence was estimated by three different approaches [9]:

- **Crude prevalence (P).** It was calculated as the total number of smear, culture or bacteriologically positive cases detected during the survey divided by the total number (n) of individuals who were screened by interview and whose both sputum results were available. Standard error (SE) was estimated as Standard deviation (SD)/√n where SD = √P (1−P). Confidence intervals (95%) of the estimates were calculated as the mean of the binomial exact ± 2SE.
- **Cluster level analysis.** Average cluster level prevalence was calculated as P_cluster level_ = ∑p_i_/c, where p_i_ is the crude prevalence in i^th^ cluster and c is the number of clusters. SE was calculated as SD/√c where SD = √[∑(p_i_−P_cluster level_)^2^/c]. Confidence intervals (95%) were calculated as the mean prevalence across clusters ± 2SE.
- **Individual level analysis.** To correct for bias introduced by incompleteness of data, individual level analysis was performed using logistic regression model with robust standard error. To include all eligible individuals in analysis, missing value imputation was undertaken for individuals: (a) without symptom screening (b) symptoms present but the result of one or both sputum specimen not available. The method accounted for between-cluster variability and uncertainty in estimating SE, under the assumption that within groups of individuals belonging to same sex, age-group and TB symptoms, data are missing at random. For imputation of missing values for each variable, starting values were assigned to missing data, which in turn was obtained from a random sample of values from individuals whose data were available. Model was then fitted with this particular variable as outcome variable and other variables as explanatory variables. This was done sequentially, in order of the proportion of data that were missing starting with variables with least missing data. Finally, a logistic regression model with smear/culture/bacteriologically confirmed TB as the outcome variable and sex, age-group and TB symptoms as explanatory variables were fitted. Newly imputed values were used as starting values for next iteration of the process which was undertaken in ten cycles in order to obtain one imputed data set. Five such data sets were imputed and the average of their prevalence was considered as final prevalence. This model takes account of clustering in the survey design, variation in the number of individuals per cluster and the uncertainty introduced by imputation of missing values when estimating the 95% CI for prevalence of PTB [9].

In six clusters where both screening tools were used, individual level analysis was also performed separately with MMR reading as an additional explanatory variable after imputing its missing values. A multiplication factor was calculated as the ratio of prevalence while using both screening tools to the prevalence using symptoms screening alone. This factor was applied to the entire study population to estimate prevalence corrected for non-screening by MMR.

Chi-square test with continuity correction was used to test the significance of differences between proportions and p-values<0.05 were considered significant.

### Study participants

A total of 71,874 individuals across 158 villages in 15 clusters were eligible for inclusion in the survey. Of them, 63,362 (88.2%) were screened by interview; 8512 (12%) could not be screened due to not being available despite repeated visits to their households. Non-participation rates were higher in males compared to females in all age groups, difference being more predominant in 15–45 years (table 1).

**Table 1:** Age and sex distribution of participants and non-participants.

Age group	Male			Female			
	No. registered	No. participated	No. not participated	No. registered	No. participated	No. not participated	P value*
15–24	9324	7506	1818	8728	8159	569	0.009
		(80.5)	(19.5)		(93.5)	(6.5)	
25–34	8542	6506	2036	7948	7446	502	0.002
		(76.2)	(23.8)		(93.7)	(6.3)	
35–44	6567	5147	1420	6181	5911	270	<0.001
		(78.4)	(21.6)		(95.6)	(4.4)	
45–54	4568	3825	743	4839	4645	194	0.32
		(83.7)	(16.3)		(96.0)	(4.0)	
55–64	3326	2973	353	3937	3766	171	<0.001
		(89.4)	(10.6)		(95.7)	(4.3)	
65+	4079	3806	273	3835	3672	163	<0.001
		(93.3)	(6.7)		(95.7)	(4.3)	
Total	36406	29763	6643	35468	33599	1869	<0.001
		(81.8)	(18.2)		(94.7)	(5.3)	

(): percentages out of numbers registered.

* for test of significance between rates of participation between males and females.

Size of eligible population varied from 3620 to 7473 in different clusters. Male to female ratio was about 1∶1 in each cluster.

## Results

### I. Prevalence of PTB based on symptom screening followed by sputum examination

Of the persons interviewed, 4681 (7.4%) were found to have symptoms. Of them, about 83% had cough of 2 weeks or more-either alone or in combination with one or more of the other symptoms- chest pain, fever, haemoptysis. The remaining 17% had one or more of the other symptoms (table 2).

**Table 2:** Frequency distribution of persons with different pulmonary symptoms.

Symptoms	No.
Cough alone	2365 (50.5)
Cough with other symptoms	1524 (32.6)
Chest pain alone	544 (11.6)
Fever alone	47 (1.0)
Haemoptysis alone	91 (1.9)
Combination of symptoms without cough	110 (2.3)
Total	4681 (100.0)

(): percentages out of total numbers with symptoms.

Previous history of ATT was present in 653 (1.0%) persons and 56 (0.09%) were currently on ATT. Of the total of 709 persons with history of ATT, 270 had symptoms at the time point of the survey while 439 (0.7% of the total interviewed) did not have symptoms.

Of 5120 persons (4681 with symptoms and an additional 439 with history of ATT) eligible for sputum examinations, spot specimen were collected from 4850 (94.7%), of which 28 were positive for AFB on smear microscopy and 65 on culture. Overnight specimens were collected from 4719 (92.2%), of which 42 were smear positive and 61 were culture positive. Overall, 51 persons were smear positive and 86 culture positive, on spot and/or overnight specimen. About 1% of spot and 1.8% of overnight specimen were contaminated.

There were a total of 110 bacteriologically positive patients. Of them, 101 were picked up through screening for presence of symptoms and an additional nine through screening for history of ATT. Among those with symptoms, 89 (88.1%) had cough with or without other symptoms while 12 (11.9%) had one or more symptoms other than cough.

Estimated point prevalence of smear positive, culture positive and bacteriologically positive PTB estimated by three analytical methods (using screening by interview for presence of symptoms) is presented at Table 3. Considering the estimates by individual level analysis as the best estimates, prevalence of smear positive, culture positive and bacteriologically positive PTB was 83 (57–109), 152 (108–197) and 196 (145–246) per 100,000 population respectively.

**Table 3:** Prevalence of PTB per 100,000 population, by method of estimation, all clusters-using screening by symptoms alone.

Type of PTB	Method of Analysis		
	Crude Prevalence	Cluster level analysis	Individual level analysis
Smear Positive PTB	81 (62–100) *N = 62959*	74 (44–103) *N = 62959*	83 (57–109) *N = 71874*
Culture Positive PTB	137 (112–161) *N = 62840* ∧	126 (85–168) *N = 62840* ∧	152 (108–197) *N = 71874*
Bacteriologically Positive PTB	175 (147–203) *N = 62841* ∧	162 (112–213) *N = 62841* ∧	196 (145–246) *N = 71874*

( ): 95% confidence intervals.

N: numbers included in analysis.

∧ : Reason for difference in numbers analyzed by three methods: for crude and cluster level prevalence, only those individuals from whom all actual data is available were considered for analysis; persons missed from screening for symptoms and persons who were eligible for sputum collection but one or both sputum specimen not collected or found contaminated on culture were excluded.

### II. Prevalence of PTB corrected for non-screening by MMR

In six clusters where both screening tools were used, there were a total of 31, 823 eligible persons of whom 28,398 (89.2%) were screened by interview and 26,429 (83.0) by MMR. Using individual level analysis, prevalence of bacteriologically positive PTB by using screening for symptoms (and ATT) was 298 (CI: 327–451) per 100,000 population; it was 389 (CI: 243–354) per 100,000 population when screening by MMR was also considered. Multiplication factor for prevalence to correct for non-screening by MMR was 1.3.

Considering the estimates by individual level analysis as the best estimates, prevalence among all persons ≥15 years of age residing in 15 clusters, corrected for non-screening by X-ray, was 108 (CI: 82–134), 198 (CI: 153–243) and 254 (CI: 204–301) per 100000 population respectively for smear, culture and bacteriologically positive PTB.

### III. Relationship of Prevalence with sex and age

Estimated crude prevalence by symptom screening alone was used to find out the relationship with sex and age. Prevalence was about six times higher among males compared to females and generally increased with age (Table 4).

**Table 4:** Prevalence (crude) of bacterologically positive PTB per 100,000 population, by age-group and sex.

	No.	Prevalence	Odds ratio
**Age group (Yrs)** *			
15–24	15602	57.7 (25.9–89.5)	1.00
25–34	13866	108.2 (62.0–154.4)	1.88 (0.77, 4.63)
35–44	10971	182.3 (115.0–249.6)	3.16 (1.37, 7.49)
45–54	8388	154.9 (83.9–225.9)	2.69 (1.08, 6.81)
55–64	6648	420.2 (288.8–551.6)	7.33 (3.31,16.7)
65+	7366	339.4 (227.6–451.2)	5.9 (2.63, 13.6)
**Sex** **			
Female	33403	50.9 (30.6–71.2)	1.00
Male	29438	315.9 (261.8–370.0)	6.23 (3.63, 10.82)

* *χ* ^2^ for linear trend = 41.4, P value<0.001.

** *χ* ^2^ = 62.7, P value<0.001 ( ): 95% confidence intervals.

## Discussion

Prevalence of bacteriologically positive PTB in persons ≥15 years of age corrected for non-screening by MMR in the entire sample of selected clusters was estimated at 254 per 100000 population, after imputing the values for missing data. Prevalence of smear positive and culture positive PTB was estimated at 108 and 198 respectively.

Prevalence was not estimated for abacillary (bacteriologically negative but radiologically active) TB, due to low specificity of diagnosis of PTB when based on single X-ray picture especially in field conditions [10]. Similarly, tools for diagnosing EPTB are too cumbersome to apply in field conditions.

A common generic protocol was used for simultaneously carried out surveys during 2008–10 at eight sites in the country including Nelamangala. However, screening by symptoms only was undertaken at five sites; MMR as additional screening tool was used in the study populations at two sites and a proportion of study population in Nelamangala. Using only symptom screening, prevalence of bacteriologically positive TB at these eight sites varied between 129 (CI: 92–165) to 399 (325–469) per 100,000 population [11]. Prevalence at three sites where both screening tools were used varied between 199 (CI: 147–251) to 391 (CI: 352–430) [11].

A number of surveys had earlier been carried out in different parts of India since mid-twentieth century. In 1956, a nation-wide survey using screening by MMR followed by sputum examination by smear and culture among those with abnormal shadow on X-ray film revealed the prevalence of bacteriologically positive PTB at 400 per 100,000 population [1]. During subsequent surveys in different geographical locations at different points of time, prevalence of bacteriologically positive TB varied between 182–1270 per 100,000 [2]. These surveys not strictly comparable due to variations in definition of symptoms, screening tools (symptoms and/or MMR), case definition and analytical methods, nonetheless revealed that TB continued to be a high burden disease in India. Male to female ratio in these surveys has been found to vary between 2∶1 to 5∶1 [2]. It was 6∶1 during the present survey.

The earlier survey in Nelamangla sub-division during 1975 was carried out in 55 villages selected by SRS method [6]. A total of 12,105 persons ≥15years of age were screened for pulmonary symptoms as well as by MMR. As in the present survey, a spot and an early morning sputum sample were collected from those with symptoms and/or any abnormal shadow on MMR. However, there were differences in age structure with higher proportion of persons in elder age groups during the present survey. We compared the prevalence at the two surveys in the age structures as they existed at given times since TB prevalence has been found to increase with age [2]. Thus increase in life expectancy per se can lead to increase in prevalence and can offset any declining trends. We compared the two surveys for crude prevalence of culture positive PTB using symptom screening only as authors of previous survey have reported only crude prevalence of culture positive PTB and that a proportion of study population during present survey was not screened by MMR. This crude prevalence was 311 per 100,000 population in 1975 and 137 (112–161) in the present survey [6]. This denotes a decline in prevalence by 56% between the two survey periods^¥^ Longitudinal studies in other areas during NTP era had revealed no significant change in prevalence [2]. Thus assuming that prevalence in the present study area remained steady up to 1990, there was 56% decline in crude prevalence between base year of Millennium Development Goals (MDGs) of the United Nations and 2009 (mid-point of present survey). This decline in Nelamangla can be attributed to overall strengthening of TB control activities due to political commitment, implementation of DOTS strategy with quality assured sputum microscopy and administration of a rifampicin based treatment regimen under observation with high rates of treatment success [3]. During the year 2010, case notification rate of PTB (new + re-treatment) under RNTCP in the Nelemangala Sub-division was 203 per 100,000 population in ≥15 years age group [unpublished quarterly case finding reports of Nelamangala Tuberculosis Unit, published data available only at district level]. This corresponds to 80% of the estimated point prevalence of bacteriologically positive PTB at 254 per 100,000 population. Prior to DOTS, TB programme performed poorly due to lack of adequate infrastructure and treatment with a self-administered regimen as in the rest of the country [12]. The progress towards MDGs from the base year of 1990 was thus far available only from Tiruvallur where three rounds revealed a decline in prevalence by about 50% between 1999 and 2006 [5]. Trends in prevalence of PTB in other Asian countries have revealed declines of 32% in China between 1990- 2000, 30% in Philippines between 1997-2007 and 67% in Indonesia between 1980-2004 [13]–[15].

A major limitation of the present survey was that mobile odelca camera based MMR unit broke down mid way and could be put back to use only after a long gap. This underlies the necessity of a back up machine. We worked out a multiplication factor of 1.3 to correct for non-screening by X-ray from the data in a subset of the eligible population in which both screening tools were used. This factor has been assumed to be homogenous across the study population which could be a limitation of this approach given that the prevalence even by symptom screening alone was higher in the six clusters where both screening tools were used compared to other clusters. Incidentally, these six clusters were located in closer proximity to an urban area and had higher population density than the rest. These six clusters were however not representative of the study area. Same correction factor of 1.3 has been estimated in Wardha district from the simultaneously carried out survey in 2008–09 (unpublished data). During earlier surveys in different parts of the country, this correction factor was found to vary between 1.5^€^ and 1.7 [16]–[19]. This variation may be attributed to variation in quality of symptom screening by field surveyors.

Another limitation could be that missing value imputation might not fully account for the missing cases in the event of higher prevalence among the absentees compared to those surveyed.

Finally, the estimated prevalence reveals that TB is still a major problem considering that much lower levels of prevalence have been observed in countries with good TB control programs in place [20]. To further reduce the disease prevalence, further strengthening of the program may be undertaken to ensure universal access to diagnosis, early detection of all incident TB patients including multi-drug resistant (MDR) patients and to achieve higher rates of treatment success. Repeat surveys if carried out in all eight recently surveyed sites after a reasonable time interval of approximately 7–8 years would provide information on future trends in different parts of the country.

^¥^decline was calculated using the formula: (P_2_−P_1_) ^*^100/ P_1_, where P_1_ and P_2_ represent the prevalence at the previous and present survey respectively

^€^correction factor of 1.5 implies that 33% of the cases will be missed if only symptom screening is employed.
